# Object-level complete coverage path planning for excavators in earthwork construction

**DOI:** 10.1038/s41598-023-40038-3

**Published:** 2023-08-07

**Authors:** Ming Yao, Xianying Feng, Peigang Li, Yanfei Li, Zhiying Peng, Ziteng Lu

**Affiliations:** 1 School of Mechanical Engineering, Shandong University, Jinan, 250061 Shandong China; 2 Key Laboratory of High Efficiency and Clean Mechanical Manufacture of Ministry of Education, Shandong University. Jinan, Shandong, 250061 China; 3 School of Information Science and Engineering, Shandong University, Qingdao, 266235 Shandong China

**Keywords:** Engineering, Mathematics and computing

## Abstract

Autonomous excavators are gradually gaining attention because they can reduce the waste of human resources and improve the efficiency. This study proposed a complete coverage path planning algorithm for autonomous excavators based on the Rotating Calipers Path Planning (RCPP) algorithm, which called the Excavator-Rotating Calipers Path Planning (E-RCPP) algorithm. This study uses boustrophedon cellular decomposition (BCD) to decompose the construction area to obtain the convex and non-convex sub-areas without obstacles, and describes a non-decomposition principle to determine whether to decompose non-convex areas that are difficult to plan. To obtain the optimal path, an adaptive spacing adjustment model which is used to adjust the spacing between parallel paths is designed. To improve the coverage rate at the corner, this study proposed a novel boundary corner turning method. The algorithm's cost function considers the path length, the number of turns, the coverage rate and the overlap rate. The Digital Orthophoto Map (DOM) of the construction area is created by Unmanned Aerial Vehicle (UAV) and cropped into three polygonal areas, the 2D top-views of the them are used for simulation experiments to verify the performance of E-RCPP algorithm, the results show that the E-RCPP algorithm has better performance when applied to the complete coverage path planning for excavator compared with the traditional RCPP algorithm.

## Introduction

Earthwork is an important part of all modern construction projects. At present, almost all earthworks are dealt by construction personnel operating large machineries in relatively harsh environments^1–3^.In order to cope with the complex working environment and improve efficiency^4^, the application of cutting-edge technologies such as robotics, sensor technology and communication technology have become the mainstream of development^5,6^. Excavators are one of the most common equipment in earthworks, which can perform a variety of tasks such as stacking, loading, cutting, leveling^7^. Unlike other transport-oriented machines such as ships, aircrafts and buses, there are two main types of task planning for excavators: object-level task planning and manipulator-level task planning^8^. The former is mainly focuses on the path planning for the excavator from the initial position to the designated construction position according to the certain strategy^9^, while the latter mainly focuses on the operation details for the excavator's components after the excavator reaches the designated working position, including the rotation of the body, the expansion and contraction of the boom and bucket rod, and the interaction between the bucket and the soil^10,11^. This study focuses on object-level task planning to achieve a complete coverage path for excavator.

At present, there are few studies on object-level task planning for excavator, especially for planning an optimal complete coverage path. Edo Jelavic et al. proposed a path planning framework for walking excavator that can generate difficult-to-plan movement plans on rugged terrain without considering all degrees of freedom (DoF) simultaneously^12^, however, this scheme does not consider the change of the construction site topography when the excavator is performing the actual earthwork. Seongcheol Woo^13^ et al. proposed a complete coverage path planning strategy for excavator based on a deep reinforcement learning method, which uses stacked convolutional neural networks to extract meaningful features from the collected images of the earthwork environment and uses a full convolutional network to map the processed features to the output task, achieving better results than the Q-learning algorithm in simulation experiments, however, this method does not consider the problems of obstacle avoidance and steering of excavator under actual working conditions, but only gives the complete coverage path in the most ideal environment. An intelligent excavator system (IES) was developed by a research team at Kyungpook University, Korea^5,8,9,14^, which developed a task planning strategy based on the earthwork characteristics and environmental constraints for the autonomous excavator, proposed a complete coverage path planning algorithm considering the cooperative problems of the excavator and the loader to verify the applicability of the planned paths, this study used an expert system-based evaluation scheme and demonstrated that the path generated by the algorithm is similar to the manually determined path. However, this strategy is based on the cell decomposition method for path planning, which does not consider the actual movement trajectory of excavator during the turning process, and the evaluation scheme is greatly influenced by the operators' personal operating habits, which makes it very cumbersome to implement. Based on the shortcomings of the above research, this study considers the influence of excavator geometry, driving characteristics and construction efficiency in the path planning, so as to provide more complete route guidance for the construction and improve efficiency and safety. The specific contributions of this study are as follows: (1) An adaptive adjustment model of the spacing between parallel paths is established based on the excavator's operation range and construction characteristics. (2) The turning problem is solved by the above-mentioned model and the driving characteristics of the excavator. (3) Based on the RCPP algorithm, an E-RCPP algorithm applicable to the complete coverage path planning for the excavator is proposed.

The remaining chapters of this study are as follows: In Chapter 2, the relevant knowledge for complete coverage path planning for excavator is described; Chapter 3 focuses on the related models and solutions for E-RCPP algorithm; Chapter 4 presents the simulation and experimental validation of the E-RCPP algorithm, analyzes the simulation experimental results; Finally, chapter 5 presents the conclusions of this paper and describes the future research plans.

## Relevant knowledge

Object-level complete coverage path planning is to plan a collision-free path for the excavator, so that the excavation scope of excavator can cover every place in the construction area along this path with high coverage rate and low overlap rate. Considering the limitation of the maximum vertical excavation depth of the excavator, when the height of the mounds to be excavated exceeds the limit, it is necessary to divide them into multiple horizontal layers and excavate each horizontal layer in the order from top to bottom^8^. Since the complete coverage path planning process is similar at each layer, this study only focus on the one of the layers. The main solutions to the complete coverage path planning problem include random coverage path planning, area decomposition method, grid based methods and spanning tree coverage^15^. This study adopts the area decomposition method to divide the horizontal levels.

### Area decomposition method

**Figure 1:**
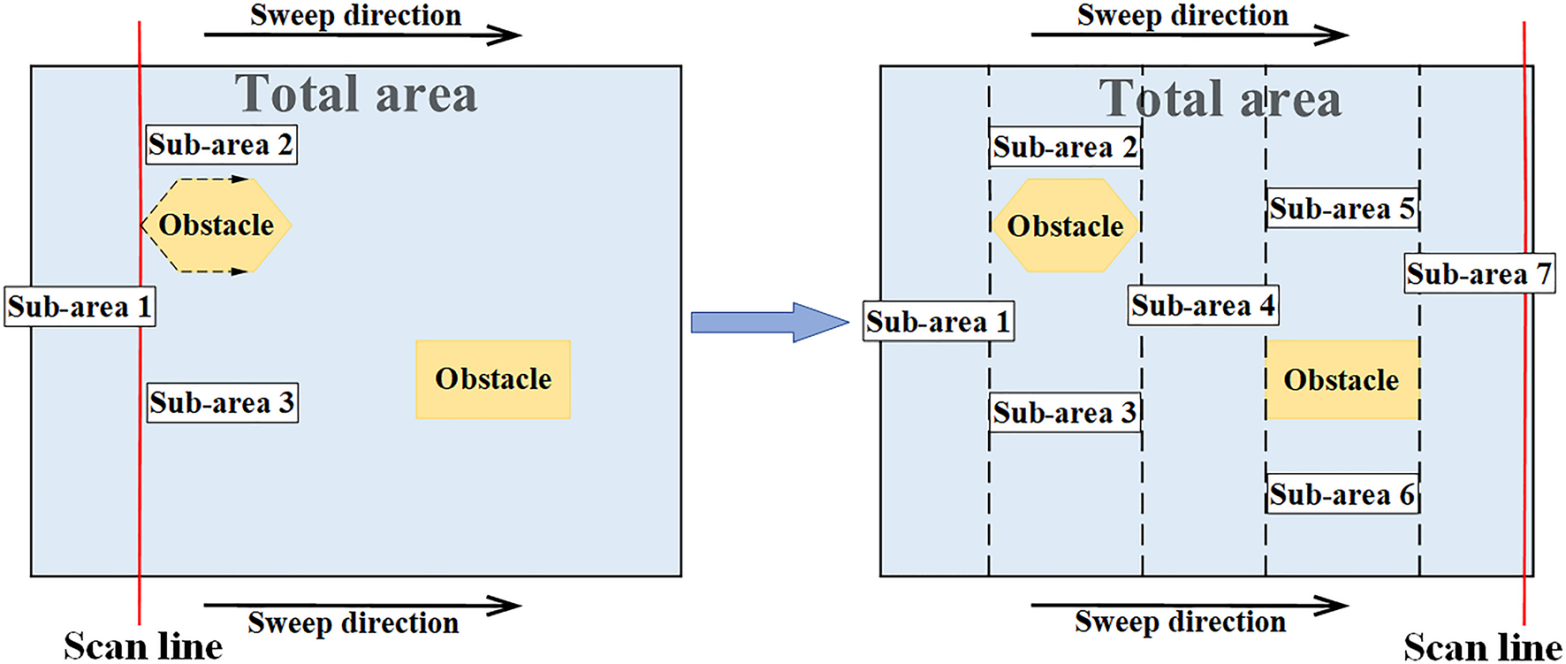
Schematic diagram of BCD.

The area decomposition method first decomposes a bounded area into a set of sub-areas that without overlapping area and obstacles, then optimizes the paths inside the sub-areas^16^, Acar et al. proposed the Morse decomposition method, which is a generalization method using the Morse function to determine the critical point of the area decomposition that can cover both polygonal and non-polygonal obstacles^17^. DH Kim et al. proposed a complete coverage path planning algorithm for mining robots based on trapezoidal area decomposition, which is a complete algorithm for mobile robots based on exact area decomposition, but its drawback is that it will generate many unnecessary areas^18^. Choset et al^19^ proposed the boustrophedon cellular decomposition method, where the boustrophedon meaning “the way of the ox”, indicating that when an ox drags a plow in a field, it walks in a straight line until it reaches the end of the field, then turns around and follows a new straight line adjacent to the previous path until it has plowed the entire field. This method is essentially a generalization of trapezoidal decomposition. By decomposing the area into cells, it allows the robot to cover each cell and thus the entire area by back-and-forth boustrophedon motions. As shown in Fig. 1, where a given target area is scanned in a certain defined direction (generally using the direction that can generate fewer sub-areas), when an obstacle is scanned, the area is decomposed according to the shape of the obstacle. The BCD is a very common method at present. Zhou et al.^20^ applied the BCD to the detection of the mining area, realized the decomposition of the mining area map, and used the neural network for path planning, so as to obtain an optimized complete coverage path. Although the area decomposition based on BCD method is widely used, this method still has some disadvantages, such as when the shape of obstacles in the area to be decomposed is complex, the decomposition will produce many trivial non-convex sub-areas, which is very unfriendly to the subsequent path planning, so in the process of practical application, the BCD needs to be improved according to the actual operational requirements.

### Path planning

**Figure 2:**
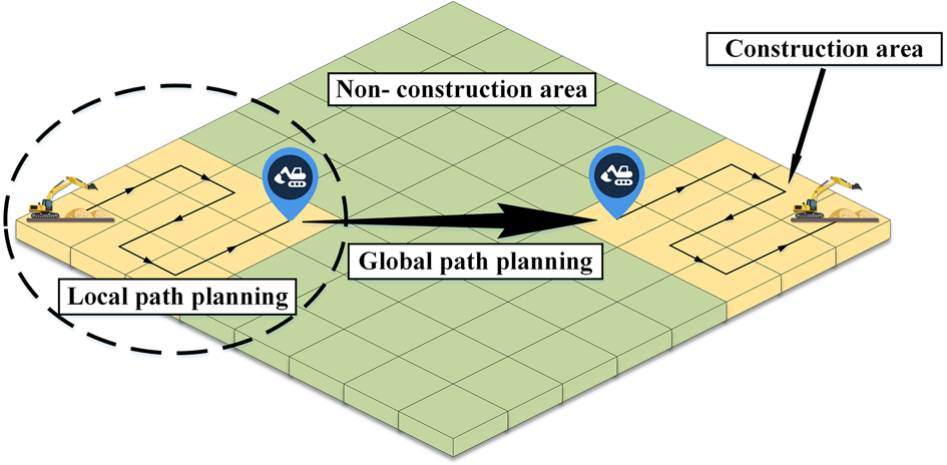
Path planning of excavator in earthwork.

The goal of path planning is to find a collision-free optimal path from the starting point to the target point in an environment with obstacles according to certain evaluation criteria^21^. According to the construction state of the excavator, their path planning can be divided into global path planning and local path planning. The global path planning means that the excavator moves from one construction area to another construction area, while the local path planning refers to the path planning when the excavator performs earthwork operations in a certain construction area. Global path planning emphasizes planning from point-to-point, generally takes the shortest driving path length, least energy consumption or the shortest driving time as evaluation criteria, while local path planning emphasizes the path to achieve complete coverage of the construction area. The object-level complete coverage path planning for excavator in this study belongs to local path planning. As shown in Fig. 2, the excavator moves between different areas through global path planning, then completes the construction of each area through local path planning, which is the entire object-level task planning process of the excavator.

At present, the paths currently used for complete coverage planning are include zigzag path, inner spiral path, and back-and-forth path (BFP). BFP is a set of parallel paths with certain spacing and end to end. Since BFP can adapt to complete coverage path planning in any convex area and is simple to calculate, therefore, this study is based on BFP to perform path planning. However, what has been verified in the current research is that the problem of finding the optimal path in an area using BFP will generate an infinite number of solutions, the problem of finding the optimal path among them is actually an NP-hard problem. Guastella performs complete coverage path planning for convex polygons by using BFP perpendicular to the positive directions of the X and Y axes, which obtains paths with fewer turns, but not optimal paths^22^. The most popular method to solve this problem is the RCPP proposed by Vasquez-Gomez^23^, which is analogous to measuring the width of a polygon using calipers. The RCPP algorithm combines the connected paths between each convex area with the complete coverage paths inside the convex areas. The optimality is defined in terms of the time required for the UAVs to follow this combined path, which is proved by mathematical method.

## Object-level complete coverage path planning strategy

Complete coverage path planning for excavator in earthworks has the following characteristics: (1) Excavator working with buckets in different positions for digging and filling operations can cause significant changes in the geometry of the surroundings. The phenomenon that the excavator is surrounded by slopes or obstacles and cannot be moved elsewhere is called “isolation”. (2) There are many obstacles at the construction areas, so the problem of obstacle avoidance needs to be considered; (3) The number of turns should be minimized due to the inconvenience of the excavator in turning. Because of the above factors, the complete coverage path planning for excavator is actually a challenging task.

### Decomposition of construction area

The sub-areas obtained based on the BCD can be classified into convex and non-convex areas according to their shapes, for convex areas it can be directly performed to path planning, but for the many trivial non-convex areas generated by the BCD^16^, considering the inconvenience of turning and the isolation problem that may be encountered, the traditional method is to decompose them into convex areas. But in fact, due to the irregularity of the shape of obstacles, it is unrealistic to decompose each non-convex area in detail to obtain idealized convex areas, which may even lead to an increase in the number of turns and seriously affecting the construction efficiency.

**Figure 3:**
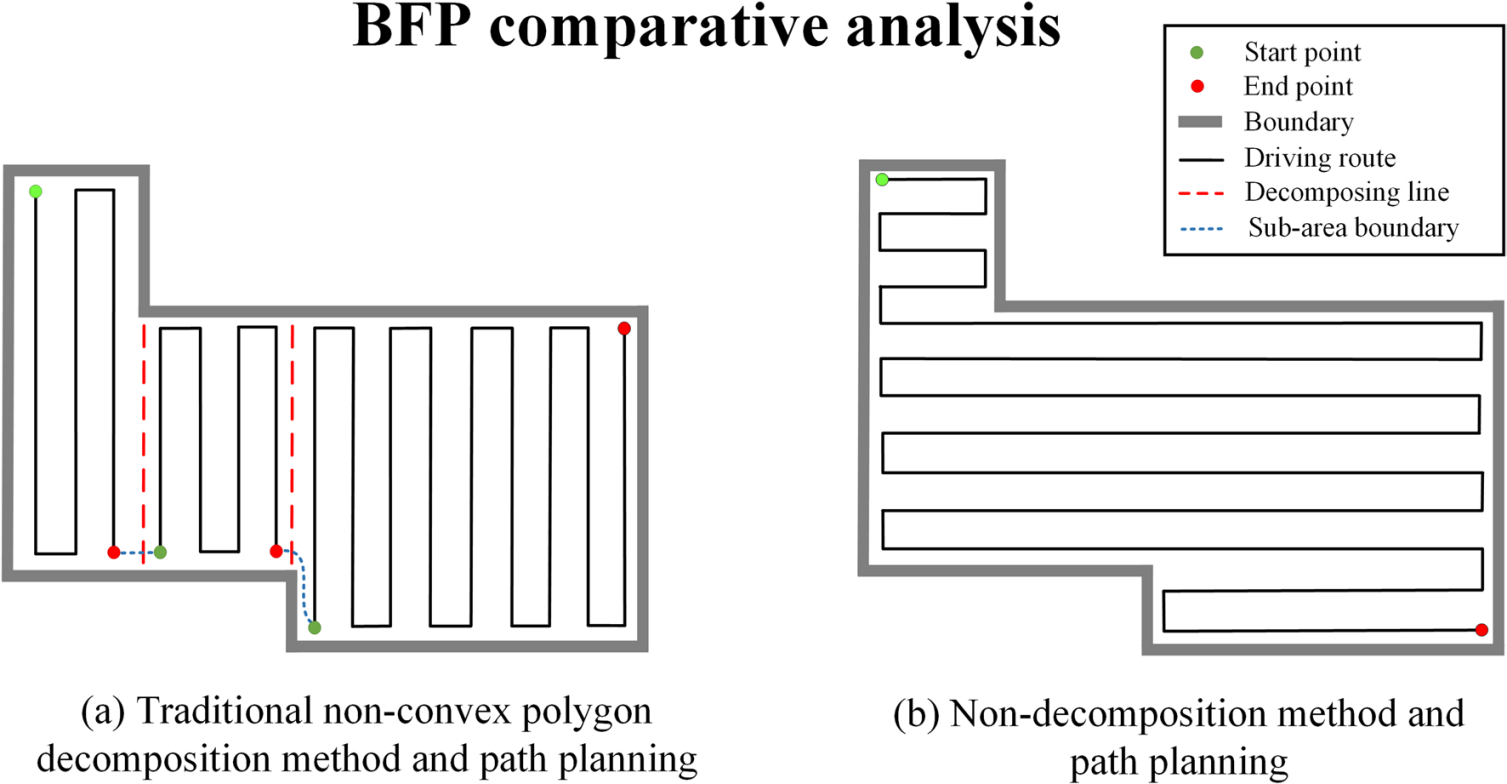
Comparison of the complete coverage path corresponding to the non-decomposition method and the traditional area decomposition method.

Considering the construction scope of the excavator, the safe construction distance and the characteristics of its own body shape, many non-convex areas can also be adequately covered by simple BFP, the effect is even better than the case of decomposing them into convex areas, as shown in the Fig. 3, the path in Fig. 3b has fewer turns than Fig. 3a. Therefore, this study propose a non-decomposition principle for the special non-convex areas: If the non-convex areas can be completely covered by the excavation scope of excavator along a BFP with spacing between $[{L}_{min}, {L}_{max}]$, and no “isolation” occurs at the end of the path, the non-convex area will not be decomposed. Here ${L}_{min}$ and ${L}_{max}$ are the minimum and maximum spacing of the BFP, respectively, they are determined by the size of the excavator.

### Excavator model building

**Figure 4:**
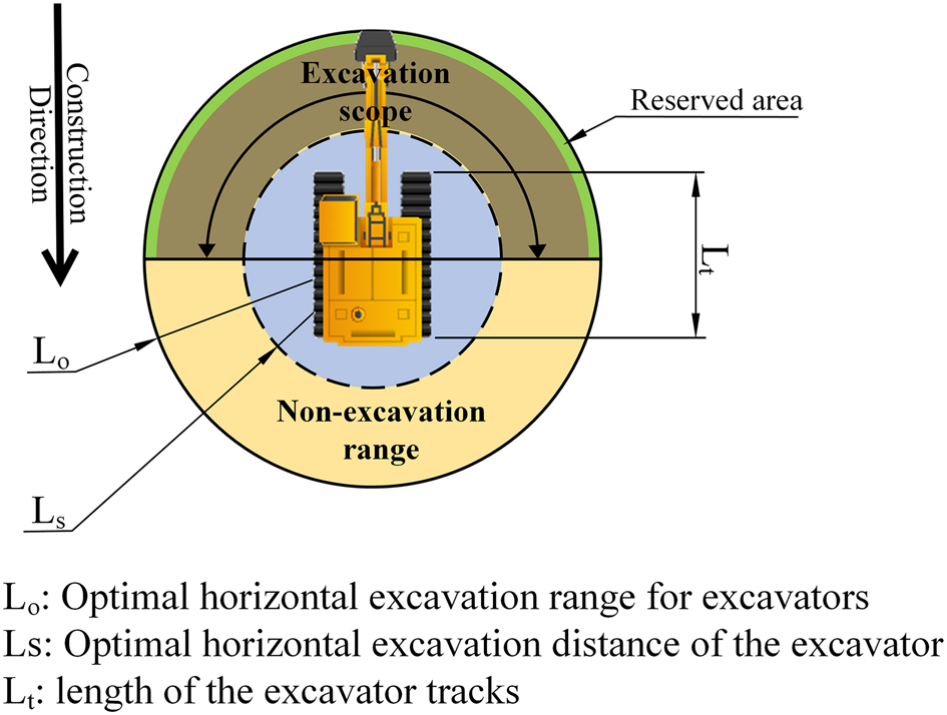
Construction model of excavator.

When the excavator is working, the best horizontal construction distance, the safe construction range and the change of the actual geographic environment of the construction areas should be considered comprehensively, Jongwon Seo et al. developed the excavator construction model shown in Fig. 4 based on the above consideration, where ${L}_{t}$ is the length of the excavator tracks, ${L}_{s}$ is to ensure the safe construction and the temporary storage of soil in the bucket limited range, ${L}_{o}$ is the optimal horizontal excavation distance. Since the excavator performs earthwork construction by moving in the opposite direction, and the backhoe may perform a specific task using different moves^24^, the excavation scope of the excavator is generally limited to 180°.To avoid the outer edge of the bucket from disturbing the soil while digging and thus causing the soil to fall back into the excavation pit, a reserved area is also provided.

**Figure 5:**
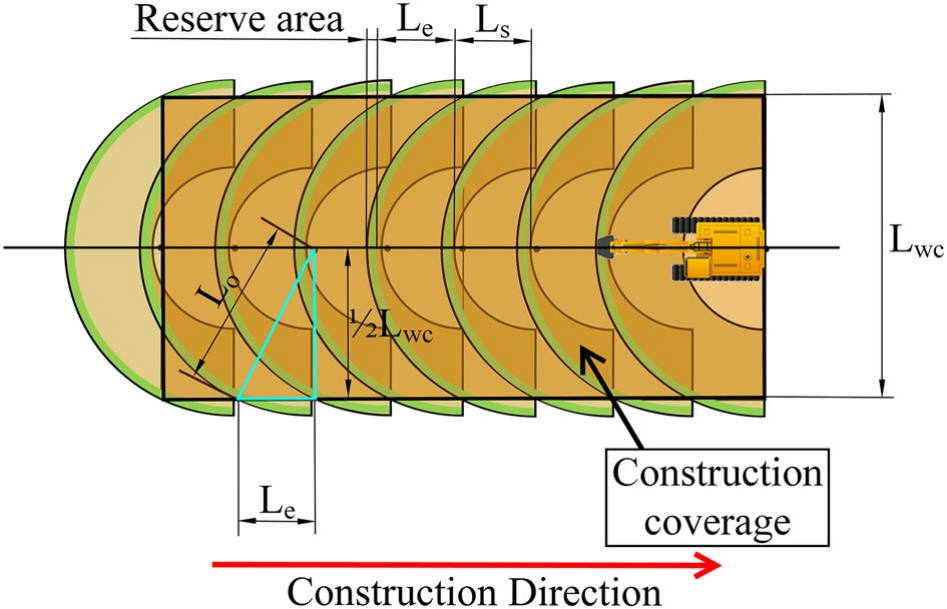
Coverage width of the construction path of the excavator.

According to the established construction model of the excavator to determine the distance between adjacent construction points and the coverage width of the construction path, ensure the continuity of the construction path coverage, as shown in Fig. 5, the distance between two adjacent construction points, ${L}_{e}$, should be slightly less than the difference between the optimal horizontal excavation distance ${L}_{o}$ and the safe construction distance ${L}_{s}$, the specific correspondence is:

1 $$L_{e} = L_{O} - L_{S} - {\text{Reserve area}}$$

The coverage width of the excavator along the path, ${L}_{wc}$, should be slightly less than twice the optimal horizontal excavation distance, ${L}_{0}$. The parameters ${L}_{0}$,${L}_{e}$,${L}_{wc}$ have the following correspondence:

2 $$L_{o}^{2} = L_{e}^{2} + \frac{1}{4}L_{wc}^{2}$$

**Figure 6:**
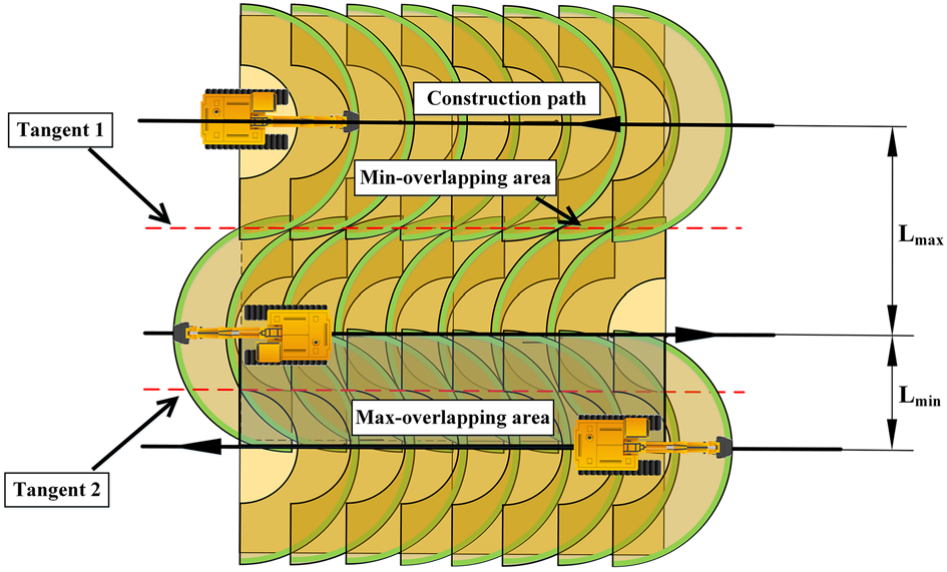
Spacing between adjacent parallel paths of excavators and overlapping areas.

In order to improve the coverage rate and reduce the overlap rate of the construction paths, and at the same time to ensure that the spacing between adjacent parallel paths is within a safe and reasonable range interval, as shown in Fig. 6, tangent1 is the common tangent of the coverage area of two adjacent parallel paths, tangent2 is the common tangent of the safe construction area of two adjacent parallel paths, the maximum spacing between two adjacent parallel paths, ${L}_{max}$, is equal to the coverage width of the excavator construction path, the minimum spacing, ${L}_{min}$, is equal to the diameter of the safe construction area, based on this spacing interval and the geometry of the construction area to establish the parallel path spacing adaptive adjustment model. From Fig. 6, it can be seen that when the spacing of the parallel paths is changed, the overlap rate of the two adjacent paths is also changed. There is a correspondence between the parallel path spacing and the path overlap rate as follows:

3 $$\left\{ {\begin{array}{*{20}l} {L_{init} = L_{wc} - D_{init} } \hfill \\ {L_{min} < L_{init} < L_{max} } \hfill \\ {D_{min} < D_{init} < D_{max} } \hfill \\ \end{array} } \right.$$

In Eq. (3), ${L}_{init}$ and ${D}_{init}$ are the initial spacing and initial width of the overlapping areas between two adjacent parallel paths in the complete coverage path, ${D}_{min}$ and ${D}_{max}$ are the minimum width and maximum width of the overlapping area. It is worth noting that since the coverage area of the construction paths in this study is a combination of a rectangle and a sector, therefore, the spacing between two adjacent parallel paths is negatively correlated with the width of the overlapping area within a specific interval, the minimum width of the overlapping area of the adjacent paths is 0.

**Figure 7:**
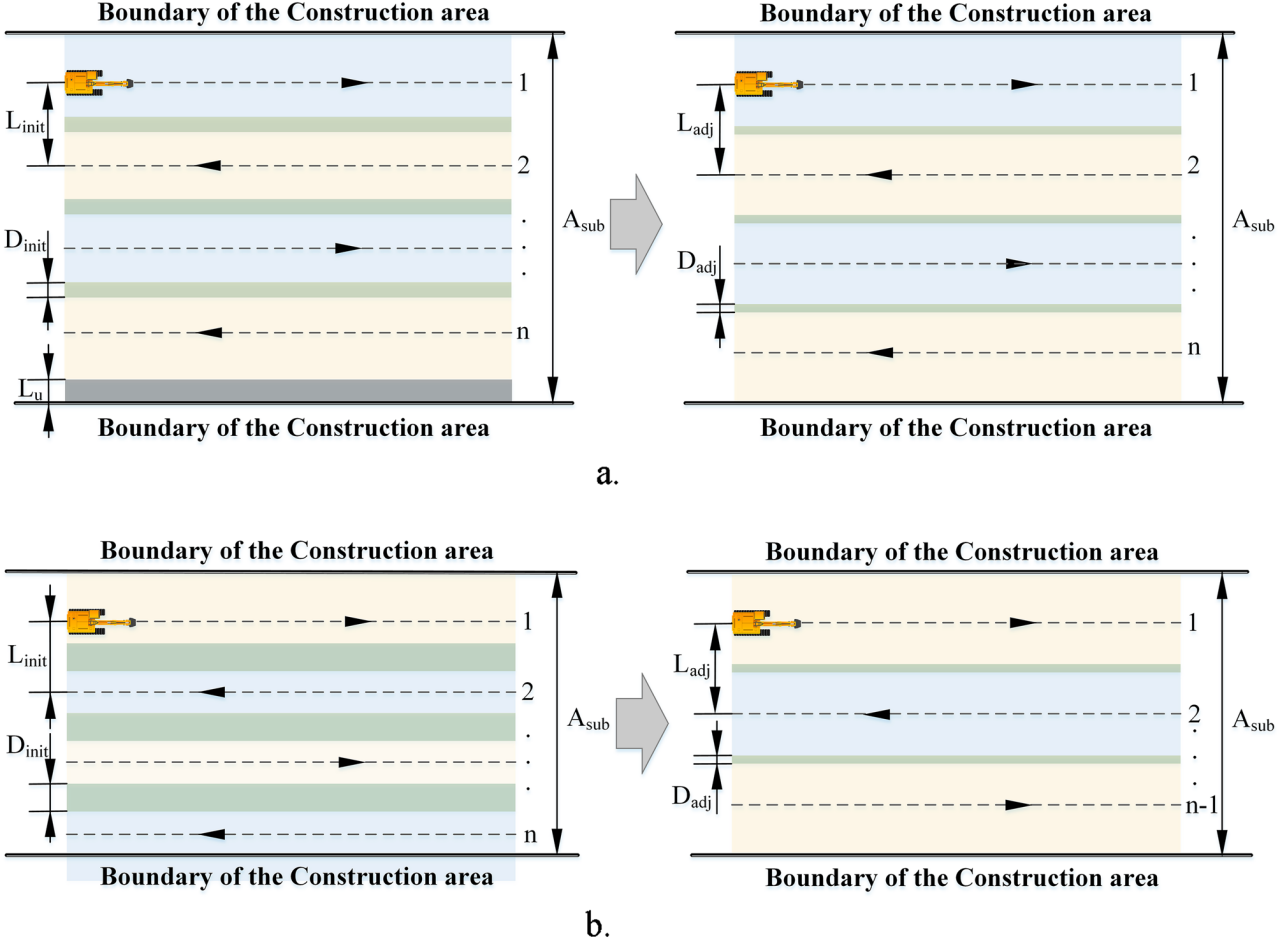
(**a**) Spacing adjustment without changing the number of parallel paths (**b**) Spacing adjustment in case of reducing the number of parallel paths.

As shown in Fig. 7, Fig. 7a shows the spacing adjustment without changing the number of parallel paths, Fig. 7b shows the spacing adjustment in case of reducing the number of parallel paths. Based on the spacing adaptive adjustment model to determine the optimal spacing for eliminating the uncovered area, the width of the uncovered area can be calculated by the following equation:

4 $$L_{{\text{u}}} = A_{sub} - (L_{wc} - D_{init} )n_{i}$$

where ${L}_{u}$ is the width of the uncovered area, ${A}_{sub}$ is the maximum width of the construction area perpendicular to the direction of the parallel paths, and ${n}_{i}$ is the number of parallel paths in the ${i}^{th}$ group of BFPs. Before making adjustments to the path spacing, it is first necessary to determine whether the number of parallel paths need to be reduced. When the total width of the overlapping area is greater than the coverage width ${L}_{wc}$ along the path, that is:

5 $$(n_{i} - 1)D_{init} > L_{wc}$$

At this time, the number of parallel paths can be reduced by one, i.e., ${n}_{i}={n}_{i}-1$, and the width of the overlapping area of two adjacent parallel paths after spacing adjustment is:

6 $$D_{init\_adj} = D_{init} - \frac{{L_{init} }}{{{\text{n}}_{{\text{i}}} - 1}}$$

Repeat the above steps until the total width of the overlapping area is less than the ${L}_{wc}$. Then determine whether the spacing between two adjacent parallel paths exceeds the maximum spacing limit, if the following inequality holds:

7 $$L_{init} + L_{dec} < L_{{{\text{max}}}}$$

The spacing between two adjacent parallel paths is adjusted to:

8 $$L_{adj} = L_{init} + L_{dec}$$

Here ${L}_{dec}$ is the increased distance of spacing and it can be expressed as:

9 $$L_{dec} = \frac{{L_{u} }}{{n_{i} - 1}}$$

If the above inequality (8) does not hold, a new parallel path is added, i.e.,${n}_{i}={n}_{i}+1$, and the spacing between two adjacent parallel paths is adjusted to:

9 $$L_{adj} = L_{init} - \frac{{L_{wc} - L_{u} }}{{n_{i} }}$$

**Figure 8:**
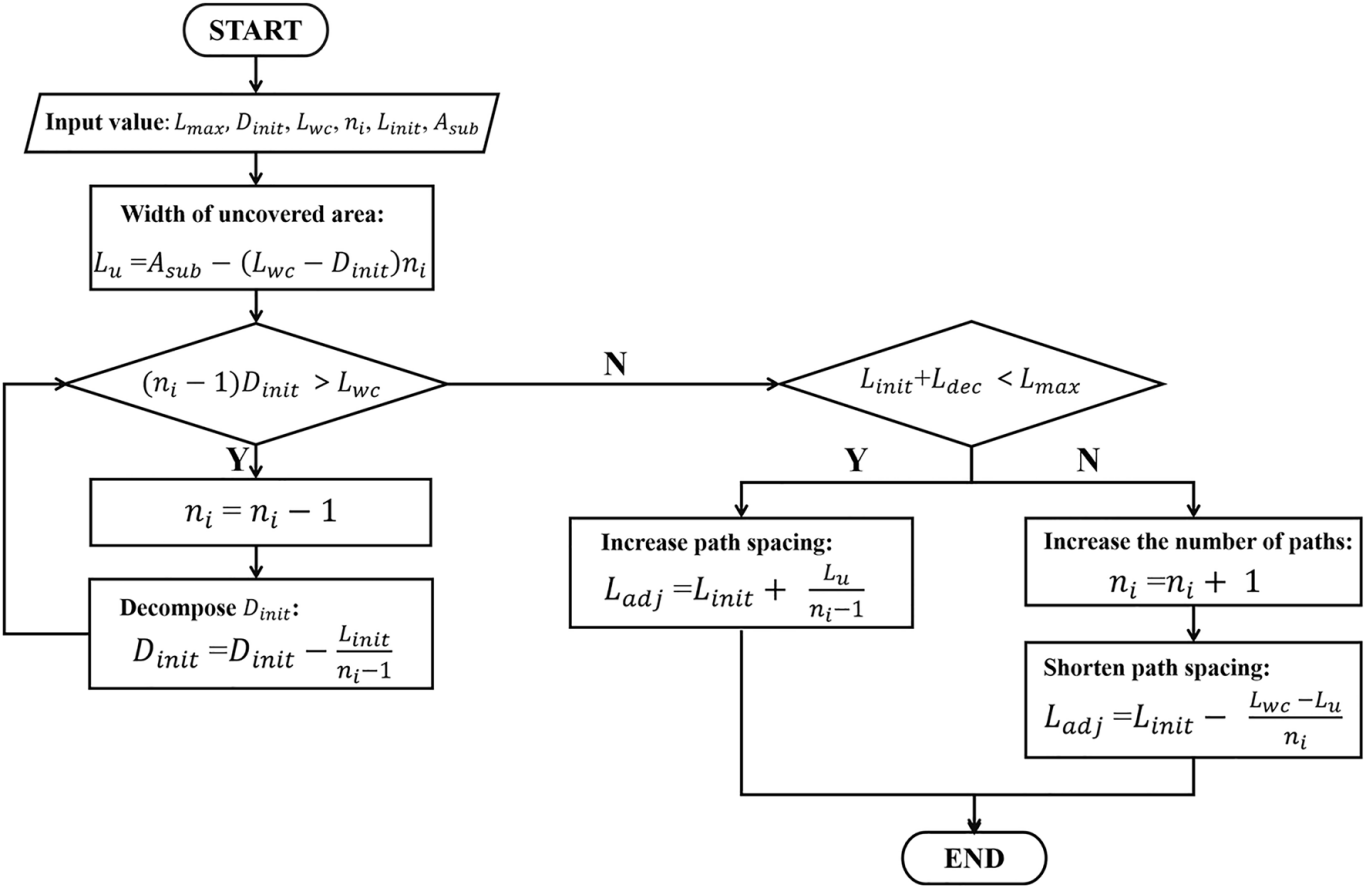
Adaptive adjustment process of the spacing for parallel paths.

It can be seen from the Fig. 7 that the width of the overlap area decreases significantly after the spacing adjustment. The specific process is shown in Fig. 8.

### Corner problem

Unlike mobile machines such as UAVs, most excavators have inconvenience in turning. Therefore, when the excavator encounters the boundary of the construction area during driving, it needs to turn around to avoid cross-border or collision early. The excavators commonly used in earthwork are crawler excavators, this type of excavators use the speed difference between the inner and outer crawler to realize the turning operation, so a suitable turning radius is generally required, which is important to adequately cover the corners. Therefore, this paper proposes a turning strategy of the excavators at the corner of the construction area to effectively connect the parallel paths of each section.

**Figure 9:**
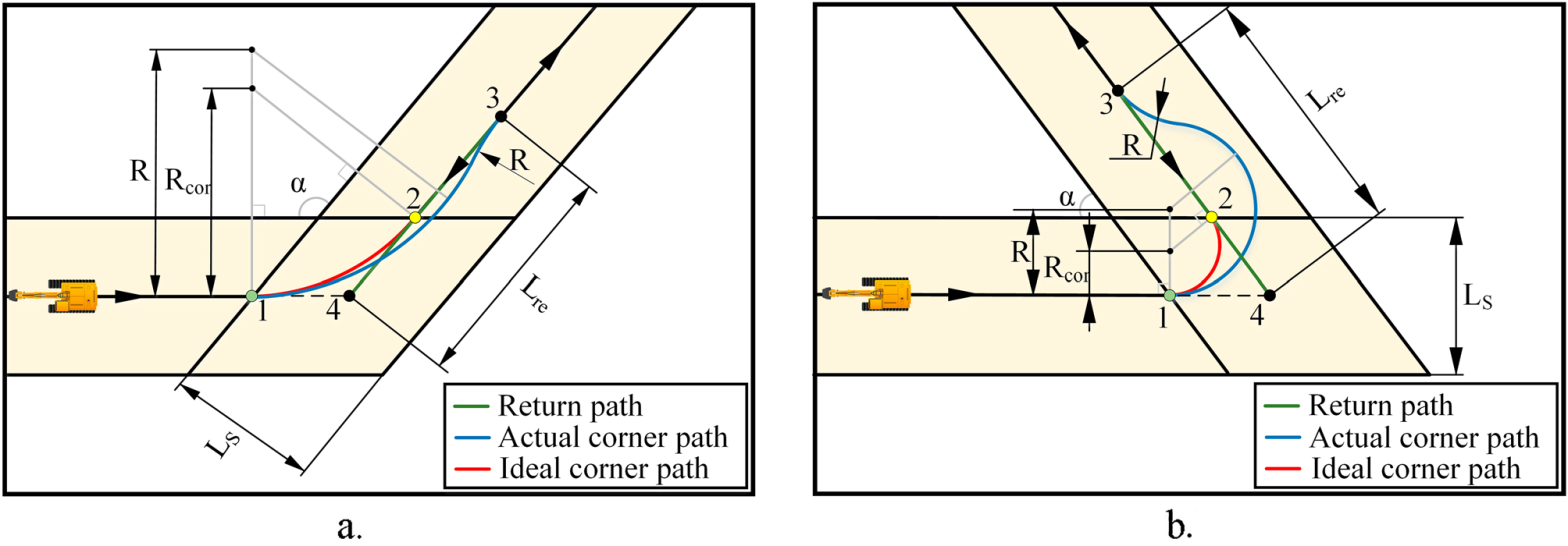
(**a**) Turning when the angle between the adjacent boundaries at the corner is obtuse. (**b**) Turning when the angle between the adjacent boundaries at the corner is acute.

Figure 9 shows the turning operation of the excavator at the corner of the construction area, where Fig. 9a shows the turning situation when the angle, *α*, of the adjacent boundary is obtuse, and Fig. 9b shows the situation when the *α* is acute. When the excavator reaches point 1, the excavator stops the earthwork operation and passes through the corner area with a certain turning radius. The specific turning strategy depends on the relationship between the radius of the common tangent circle of the adjacent boundary at the corner,${R}_{cor}$, and the minimum turning radius of excavator,$R$. The size of ${R}_{cor}$ can be calculated from the geometric relationship by Eq. (11).

11 $$R_{cor} = \left\{ {\begin{array}{*{20}l} {\frac{1}{2}L_{s} \tan^{2} \alpha - \frac{\sin \alpha }{{2\cos^{2} \alpha }}L_{s} + \frac{1}{2}L_{s} } \hfill & {\alpha \ge 90{^\circ }} \hfill \\ {\frac{{L_{s} (1 - \sin \alpha )}}{{2\cos^{2} \alpha }}} \hfill & {\alpha { < }90{^\circ }} \hfill \\ \end{array} } \right.$$

If ${R}_{cor}$>*R*, the excavator can directly follow the arc trajectory of the common tangent circle to reach point 2. If ${R}_{cor}$<R, the excavator will follow its own minimum turning radius to turn. At this time, the excavator will reach point 3 behind point 2 . In order to ensure sufficient coverage at the corners of the construction area, the excavator adopts a backtracking mechanism, that is, when the excavator reaches point 2 along an arc with the radius of ${R}_{cor}$, the excavator does not drive in the direction of point 3 next, but reverse back to the backtracking point 4, and start the earthwork operation from here. Similarly, when the excavator reaches point 3 along an arc with the radius of *R*, it also performs a reversing operation and returns to point 4, then starts the earthwork operation from here. The length of the backtracking path is ${L}_{re}$. Since the excavator needs to drive within the limits of the safe area, in order to improve the coverage rate, the backtracking point 4 is often located on the safe boundary.

After obtaining the boundary information of the construction area, it is necessary to shrink the boundary inward by a certain distance. The boundary after shrinking is called the safe boundary, which is set as $\frac{1}{2}{L}_{wc}$. On the one hand, it provides a turning space for the excavator after it drives to the border, on the other hand, it ensures the safety and efficiency of the construction in the close proximity of the boundary. The start point and end point are set on the safe boundary, and the spacing of parallel paths is determined by the spacing adaptive adjustment model. A series of waypoint commands are sent to guide the excavator along the BFP to drive to each work station in turn. When the excavator drives along a straight line to a turning point close to the safe boundary, i.e., point 1 in Fig. 9, the control system calculates the turning path and sends corresponding commands to the excavator based on the position of the excavator and the surrounding boundary information. The excavator turns into the adjacent parallel path and continues to construction until it reaches the end point. Through the above strategy, generating a complete coverage path that satisfies the driving and construction conditions is guaranteed.

### E-RCPP

Although the RCPP has achieved notable results in complete coverage path planning for convex areas of UAV, its direct application in the field of excavator earthworks is limited: (1) Due to the limitation of the distance between the adjacent parallel paths of the crawler excavator, too many turns will affect the overlap rate and length of the path, the RCPP only uses the Euclidean distance for path selection without considering the influence of the number of turns on the optimal solution of the path; (2) Unlike the UAV, which has the characteristic of being able to cross the boundary of its working area, the construction of the excavator is based on a closed environment with boundary constraints; (3) For areas that have completed construction, the excavator cannot achieve repeated traversal because the changes of the terrain during the construction process are significant, which is different from the UAV can be achieved multiple repeat traverses of an area; (4) UAV can work while driving, but the excavator only performs when the fuselage is stationary, which results in the excavator spending much more time in the work situation than the driving between the construction areas. Based on the above points, this study proposes an E-RCPP algorithm. It only uses the A* algorithm, which is a heuristic path planning algorithm for generating a shortest collision-free connected path between a start point and a goal point, to determine the connectivity between construction areas, thus preventing the occurrence of “isolation” problems, without using the length of connected paths in construction areas as a criterion for evaluating the superiority of paths. Then a simple cost function is used to evaluate the superiority of the path. Balancing efficiency and completeness of construction are often competing for objectives for a construction project, making it difficult and challenging to find the optimal tradeoff between these two objectives^25^. As shown in Eq. (12), the cost function consists of two parts, one is the drive time term, the excavator tends to have a shorter path and fewer number of turns during driving, so this term uses the product of the number of turns and the length of the path to represent the value of drive time, and divides by the perimeter of the area to represent the complexity of the path relative to the area. The other part is the construction efficiency term, as the construction process prefers high coverage rate and low overlap rate, the ratio of overlap rate to coverage rate is used to represent the construction efficiency. The lower cost value indicates the higher path superiority.

12 $$COST = \beta \frac{{N_{t} (i) \cdot L_{SP} (i)}}{{L_{PA} (i)}} + \gamma \frac{{P_{r} (i)}}{{P_{{\text{o}}} (i)}}$$

where ${N}_{t}(i)$ represents the number of turns in the ${i}^{th}$ construction area, ${L}_{SP}(i)$ is the path length in the ${i}^{th}$ construction area, ${L}_{PA}(i)$ is the perimeter of the ${i}^{th}$ construction area, ${P}_{o}(i)$ is the coverage rate of the construction area when the excavator drives along the construction path in the ${i}^{th}$ construction area, ${P}_{r}(i)$ is the overlap rate of the excavator's construction scope in the ${i}^{th}$ construction area, *β* and $\gamma$ are the adjustment coefficients, to balance the size relationship between the two items, this study set *β* = 1,$\gamma$=100.

If the BFP generated by the complete coverage path planning algorithm contains $m$ parallel paths, the number of turns can be calculated by the following equation:

13 $$N_{t} (i) = 2m - 2$$

Coverage rate ${P}_{r}(i)$ is expressed as the ratio of the construction area ${S}_{coverage}$ that can be covered by an excavator driving along a complete coverage path to the total area ${S}_{area}$. As shown in the following equation:

14 $$P_{r} (i) = \frac{{S_{{{\text{coverage}}}} }}{{S_{area} }} \times 100\%$$

The overlap rate ${P}_{o}$ is expressed as the ratio of the area of the overlapping portion of the construction scope ${S}_{overlap}$ as the excavator drives along the complete coverage path to the total coverage area ${S}_{coverage}$. As shown in the following equation:

15 $$P_{o} (i) = \frac{{S_{{{\text{overlap}}}} }}{{S_{{{\text{coverage}}}} }} \times 100\%$$

The path generated by the complete coverage path planning algorithm is divided into many individual waypoints sent to the control system of the autonomous excavator to guide its movement. Therefore, the path length ${L}_{SP}(i)$ can be obtained by calculating the distance between two adjacent waypoints and then summing them up.

**Figure 10:**
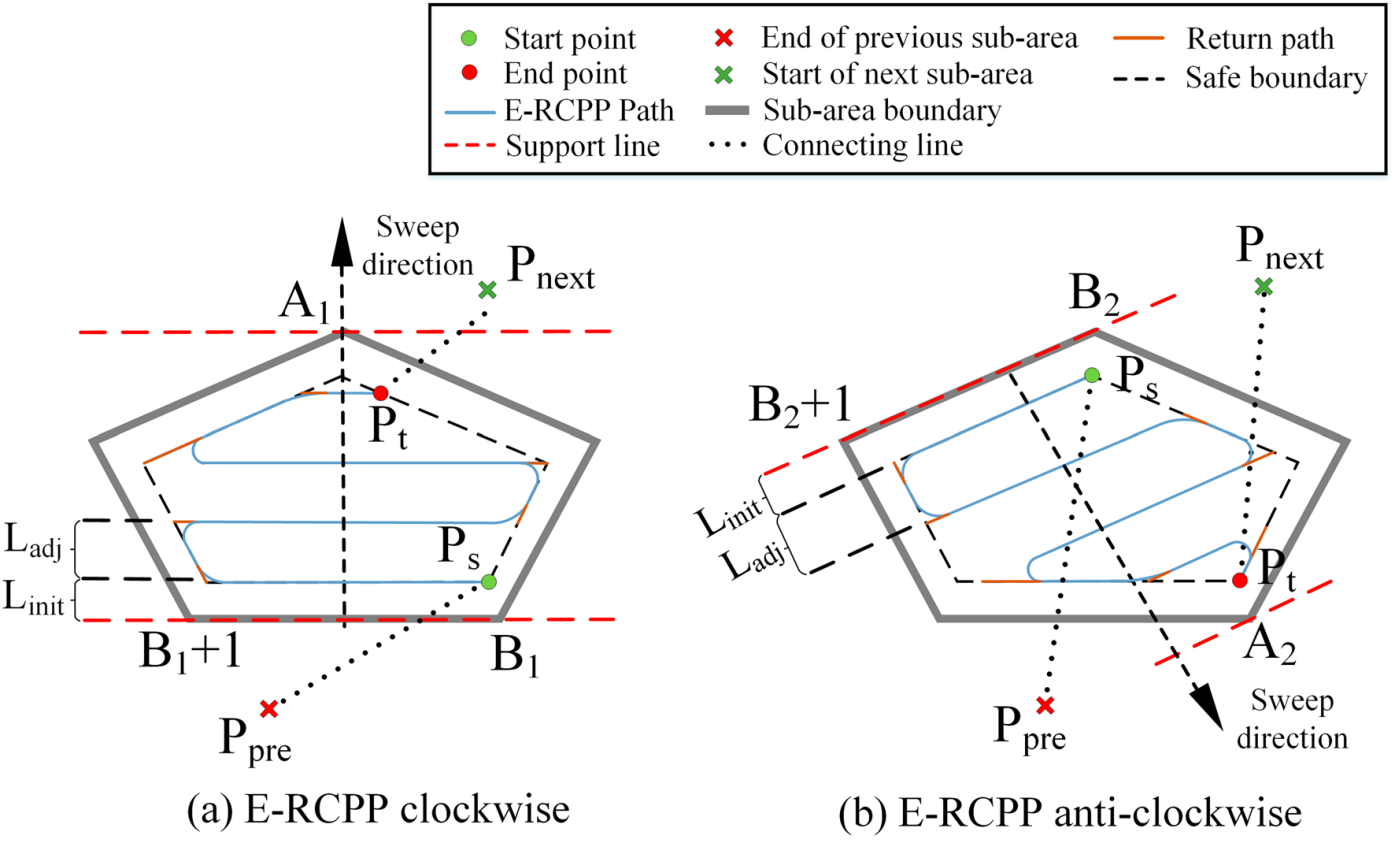
Comparison of clockwise path and anti-clockwise path generated by E-RCPP.

**Figure 11:**
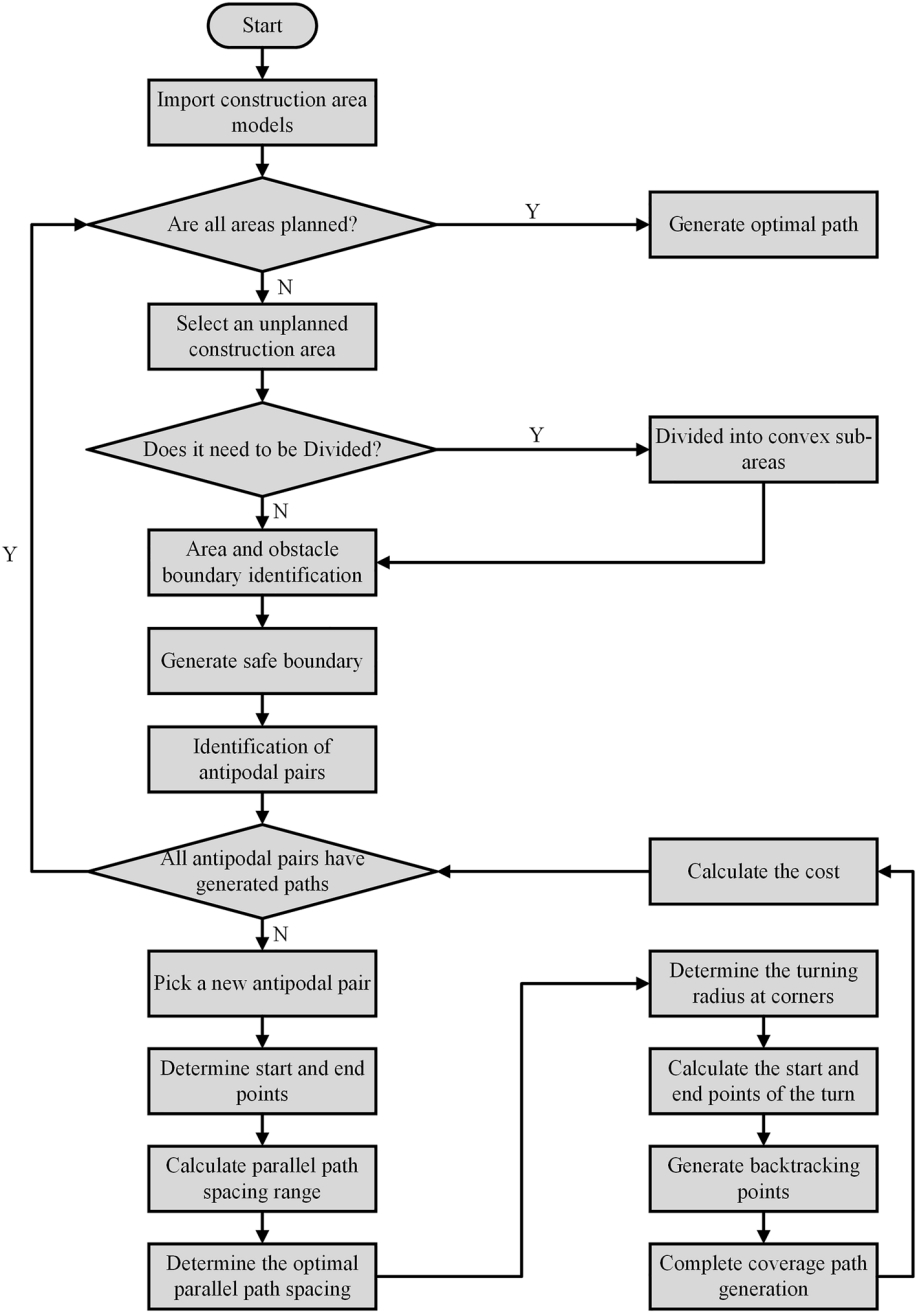
Flow chart of the complete coverage path planning algorithm proposed in this study for excavator construction.

The sub-areas obtained by the BCD are classified into convex areas and non-convex areas, where the convex area are denoted as $Q\{V,E\}$. The vertices of the convex areas in the clockwise direction are denoted as $V=\{\mathrm{1,2},\dots ,k\}$ and their edges in the clockwise direction are denoted as $E=\{\left(\mathrm{1,2}\right),\left(\mathrm{2,3}\right),\dots \left(k-1,k\right),(k,1)\}$. The area of the convex area is $S\left(Q\right)$, the coverage area of the excavator on the path $p$ is $A(p)$, If the line $L$ intersects the boundary of a convex area and lies entirely on one side of the convex area, then $L$ is called to be the support line. A pair of vertices of the convex area is called to be an antipodal pair if a pair of parallel lines can be made through this pair of vertices such that all points on the area fall between or on the lines of this set of parallel lines. All antipodal pairs are noted as $({i}_{k},{j}_{k})$. As shown in Fig. 10, the edges $({B}_{1},{B}_{1}+1)$ and $({B}_{2},{B}_{2}+1)$ are called the baselines of the convex area, and they are the first contact edges with the convex area when the support line is rotated in clockwise and anti-clockwise directions. The distal vertices of the convex area corresponding to these two baselines are respectively noted as ${A}_{1}$ and ${A}_{2}$, ${P}_{pre}$ is the end point of the previous sub-area, ${P}_{next}$ is the start point of the next sub-area, ${P}_{s}$ and ${P}_{t}$ are the start point and end point of the current sub-area respectively, ${L}_{dist}$ is the distance between the first parallel path and the corresponding baseline, its value is half of coverage width of the excavator. ${L}_{adj}$ is the spacing of adjacent parallel path adjusted by the adaptive adjustment model. Figure 10 shows the comparison of the paths generated by an antipodal pair in clockwise and anti-clockwise directions, it can be seen that they have different lengths and turns, the optimal path can be found by comparing the cost of the clockwise and anti-clockwise paths corresponding to all the antipodal pairs in the sub-area. For non-convex areas, it can be processed according to the method provided in Sect. “Decomposition of construction area”. It is worth noting that due to the non-repeatable traversal of the construction area, this figure only shows the shortest connected path between the sub-areas, not the actual connected path. Figure 11 shows the flow chart of the complete coverage path planning algorithm proposed in this study applicable to excavator.

## Experiments and analysis

### Construction scene

**Figure 12:**
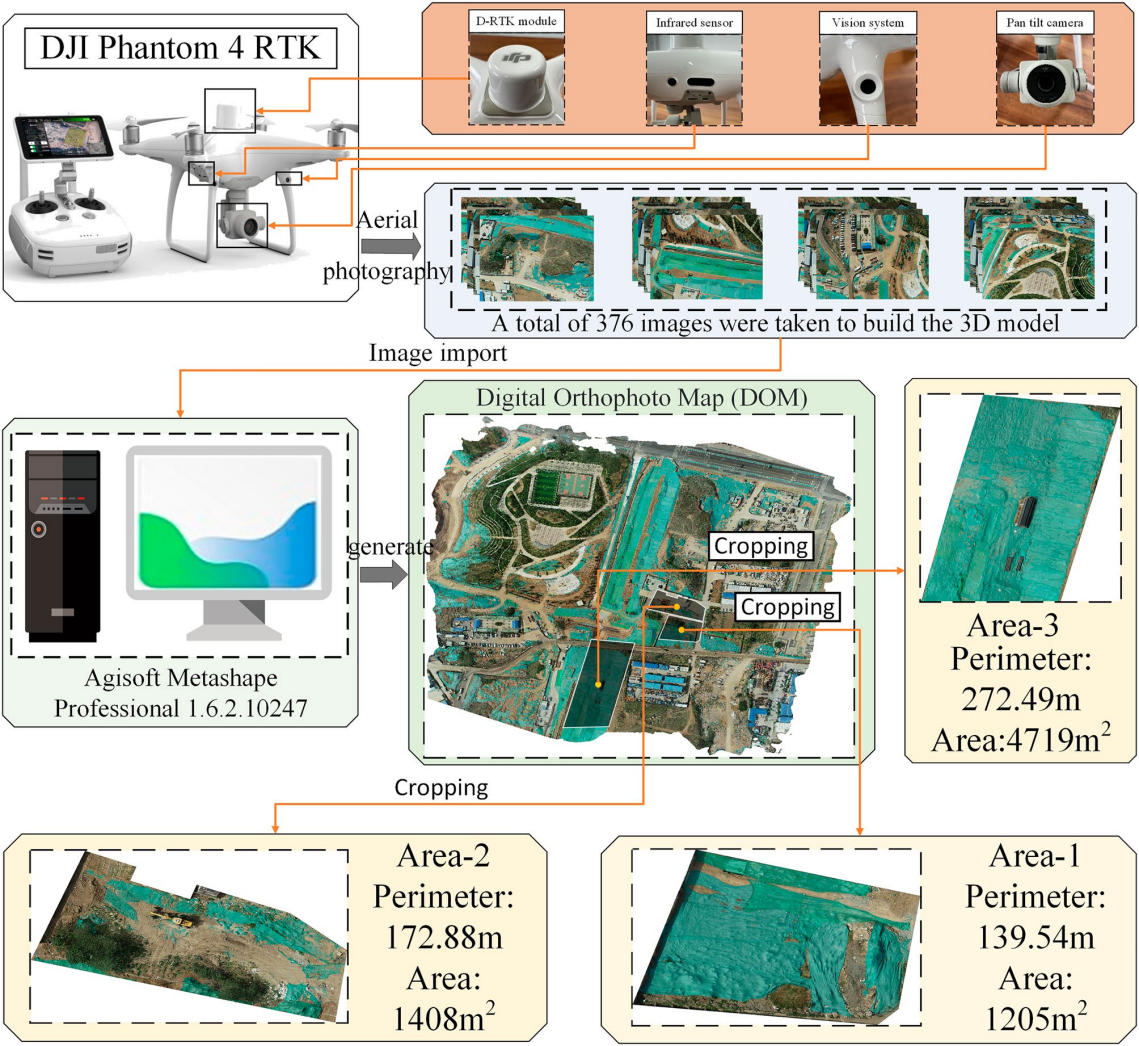
Main structure of Phantom 4 RTK and the construction process of Digital Othophoto Map.

**Figure 13:**
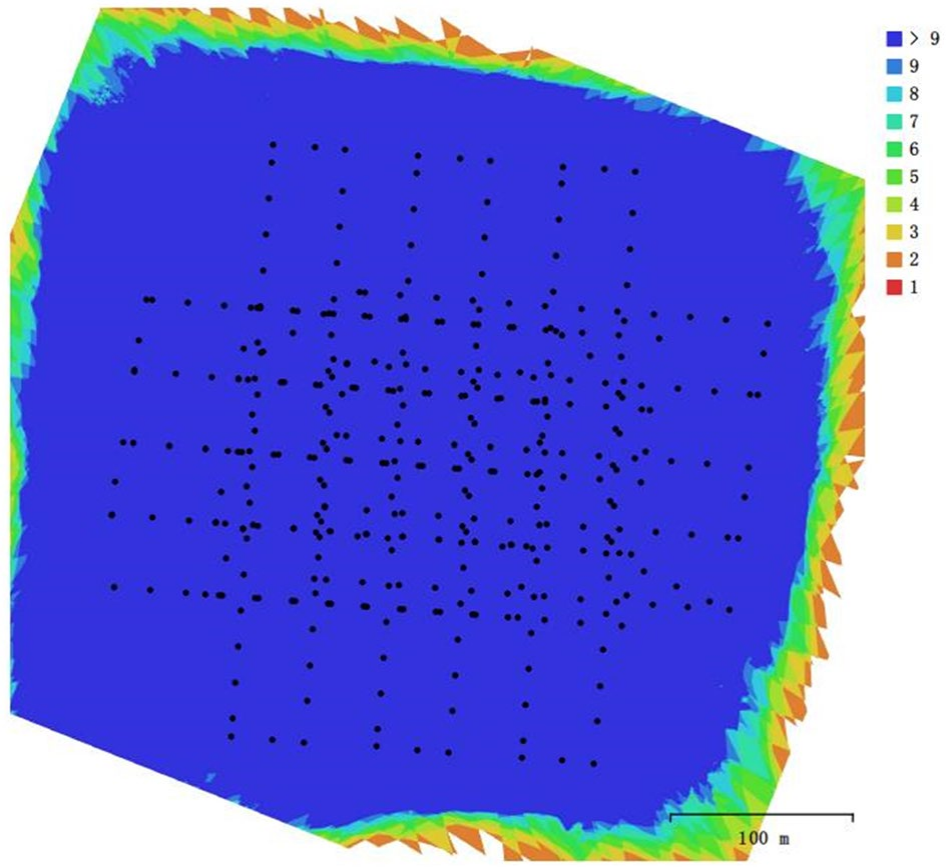
Camera location and image overlap.

**Figure 14:**
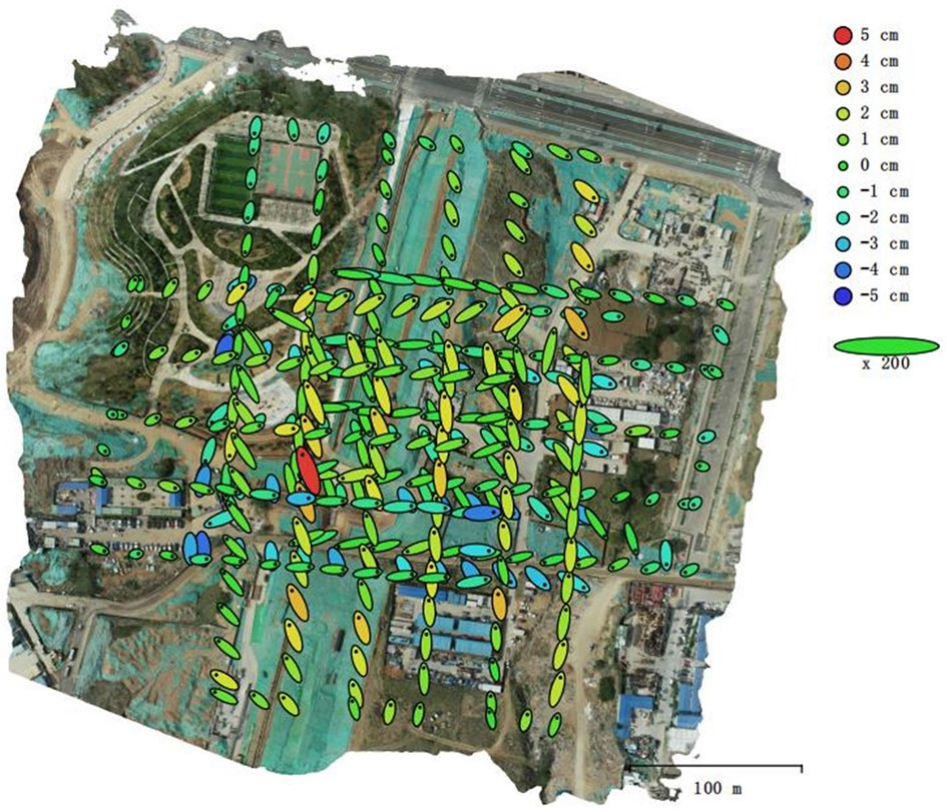
Camera location and error estimates.

To verify the effectiveness of the complete coverage path planning algorithm for excavators proposed in this study, a construction site (36°34′30.40''N, 116°58′43.44''E) was selected as the study area. Figure 12 shows the specific structure of the DJI Phantom 4 RTK and the 3D model building process for the construction site, the Digital Orthophoto Map (DOM) of the construction site established by the UAV with a five-way aerial photography, including one set of orthophoto lines and four sets of inclined lines with different heading. A total of 376 pictures were taken in this experiment, the software for 3D model reconstruction of the construction site is Agsoft Metashape Professional 1.6.2.10247. The construction areas are defined as 2D top-views over the DOM and obtained by cropping, then according to the correspondence between the sampling spacing of the generated pixel points and the actual distance to complete the coordinate conversion. The boundaries of the construction areas and the obstacles are identified to finally confirm the areas to be planned. Table 1 shows the relevant parameters for 3D model reconstruction using UAV. The camera carried by the UAV is FC6310R, its specific parameters are shown in Table 2. Figure 13 shows the location of the camera and the overlap of images. Figure 14 shows the camera's location and the estimation of its error, Z-Error (Altitude) is represented by ellipse color, X-Error (Longitude) and Y-Error (Latitude) are represented by ellipse shape, estimated camera locations are marked with black dots. The average error and the total error in each direction are shown in Table 3.

**Table 1:** The relevant parameters for 3D model reconstruction using UAV.

Parameters	Value	Parameters	Value
Number of images	376	Camera stations	376
Flying altitude	118 m	Tie points	151,153
Ground resolution	3.01 cm/pix	Projections	969,615
Coverage area	0.188 km^2^	Reprojection error	0.705 pix

**Table 2:** Parameters of Camera.

Parameters	Value
Camera model	FC 6310 R
Resolution	4864 × 3648
Focal length	8.8 mm
Pixel size	2.61 × 2.61 µm
Preliminary calibration	Yes

**Table 3:** Average camera location error.

Direction	Value/cm
X-Error	4.09029
Y-Error	4.40185
Z-Error	1.60632
XY-Error	6.00889
Total Error	6.21989

**Figure 15:**
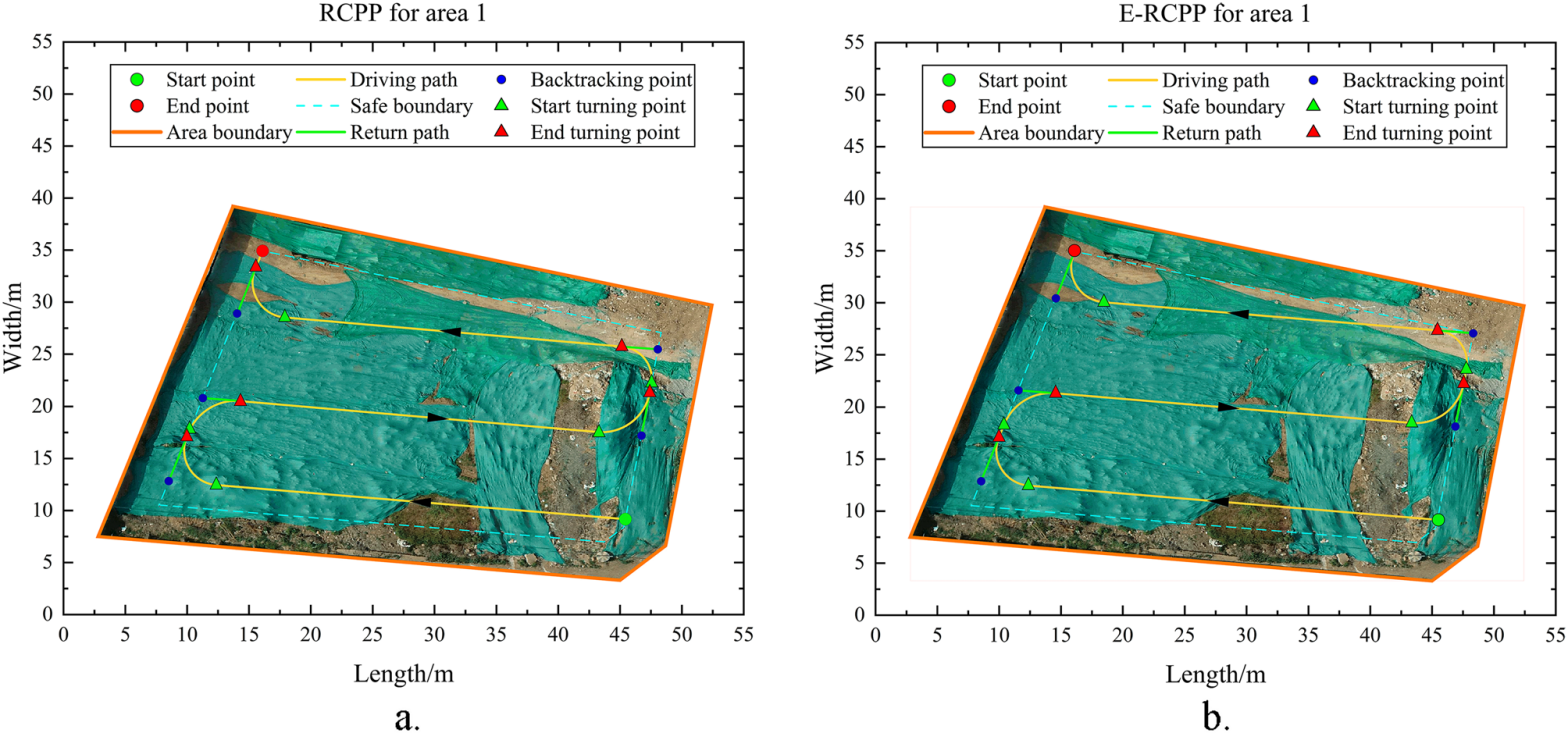
(**a**) Path generated by RCPP in area 1. (**b**) Path generated by E-RCPP in area 1.

**Figure 16:**
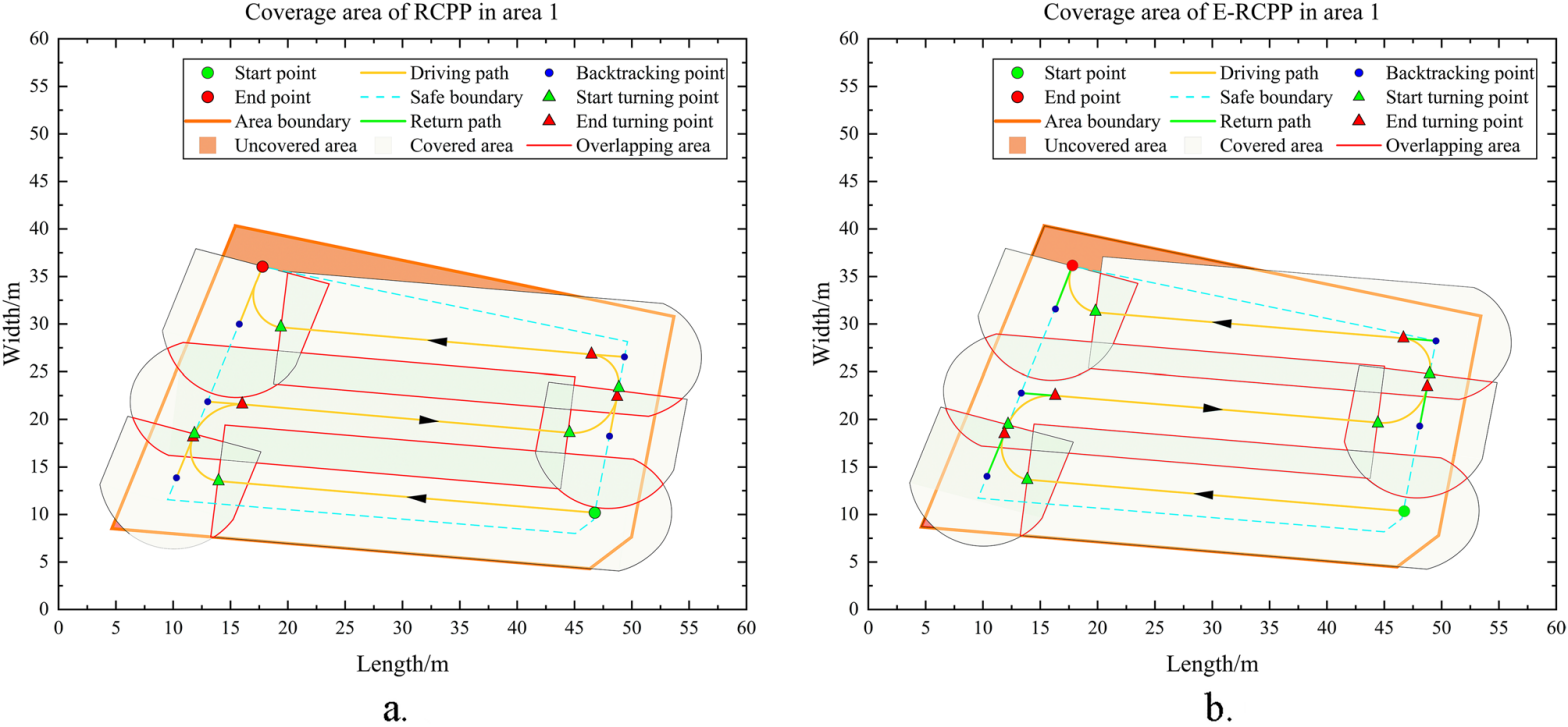
(**a**) Coverage of area 1 by the path generated by RCPP. (**b**) Coverage of area 1 by the path generated by E-RCPP.

### Excavator component parameters

The SE85-9 is a commonly used medium-sized hydraulic excavator, its component parameters provide the calculation basis for the E-RCPP algorithm. This excavator has a boom length of 3.9 m, a bucket bar length of 1.75 m and a bucket capacity of 0.35m^2^. The total length of the excavator is 6455 mm, the grounded length for transportation is 4030 mm, the overall width is 2306 mm, the track length is 2950 mm, and the track gauge is 1850 mm. When in working condition, the maximum digging distance of the excavator is 6600 mm, the optimal horizontal construction distance is 6455 mm, the maximum digging height is 7175 mm, the maximum digging depth is 5025 mm.

### Analysis of experimental results

The E-RCPP algorithm program is written based on Matlab 2020a and runs it on an intel workstation with i9-10900 CPU, 3.70 GHz and 64 GB RAM to generate object-level complete coverage paths. In order to verify the superiority of the E-RCPP algorithm proposed in this paper, the paths generated by this algorithm are compared and analyzed with those generated by the traditional RCPP algorithm. Table 4 shows the geometry parameters of the three construction areas and the comparison results of the paths generated by the two different algorithms. As shown in the Table 4, the paths generated by both algorithms have 5 turns and are very close in path length in Area 1. However, the complete coverage path generated by the E-RCPP algorithm has 2.42% higher coverage rate and 4.34% lower overlap rate than the RCPP algorithm, and the cost function is reduced by 15.10%. In Area 2, the number of turns generated by E-RCPP is 9, which is 3 times less than the RCPP algorithm, the length of the path is reduced by 4.01%, which indicates that the excavator consumes less time when driving along the path generated by the E-RCPP algorithm. Although the E-RCPP algorithm is 0.73% lower than the RCPP algorithm in terms of coverage rate, the overlap rate and the surrogate value are reduced by 25.78% and 53.61%, respectively. This optimization effect can be said to be very significant, which means higher work efficiency. In Area 3, there is not much difference in the length of the paths generated by the E-RCPP algorithm and RCPP algorithm. The number of turns is also 9 for both algorithms. In terms of coverage rate, overlap rate and cost, E-RCPP algorithm outperforms RCPP algorithm by 1.24%, 1.56% and 5.21%.

**Table 4:** Geometric parameters of the three construction areas and comparison of performance between E-RCPP algorithm and RCPP algorithm in complete coverage path planning.

Name	Algorithm	Obstacle area/m^2^	Perimeter/m	Area/m^2^	Number of turns	Path length/m	Coverage rate	Overlap rate	Cost
Area 1	RCPP	0	139.54	1205	5	152.56	94.87%	27.47%	34.426
	E-RCPP				5	152.35	97.29%	23.13%	29.229
Area 2	RCPP	0	172.88	1408	12	194.82	98.24%	41.26%	55.523
	E-RCPP				9	187.01	97.51%	15.48%	25.616
Area 3	RCPP	252.6	272.49	4179	9	454.50	98.33%	19.52%	34.862
	E-RCPP				9	454.39	99.57%	17.96%	33.046

#### Area 1

Area 1 has a flat terrain and no obstacle that affect the driving of the excavator, it is a very typical convex area, so there is no need to perform area decomposition when running the RCPP algorithm and E-RCPP algorithm, area 1 will be the most ideal and simplest experimental area in this study to make a preliminary comparison. Figure 15a and b show the paths generated by the RCPP algorithm and the E-RCPP algorithm in area 1. Figure 16a and b show the coverage area of the paths generated by the RCPP algorithm and E-RCPP algorithm in area 1. It can intuitively see that the paths planned by the E-RCPP algorithm have larger spacing, higher coverage and lower overlap, which is mainly due to the application of the adaptive spacing adjustment model proposed in Sect. “Excavator model building”. The length and number of turns of the paths planned by the two algorithms are not very different. This is mainly because the area of Area 1 is small, so it can be covered without too many parallel paths.

**Figure 17:**
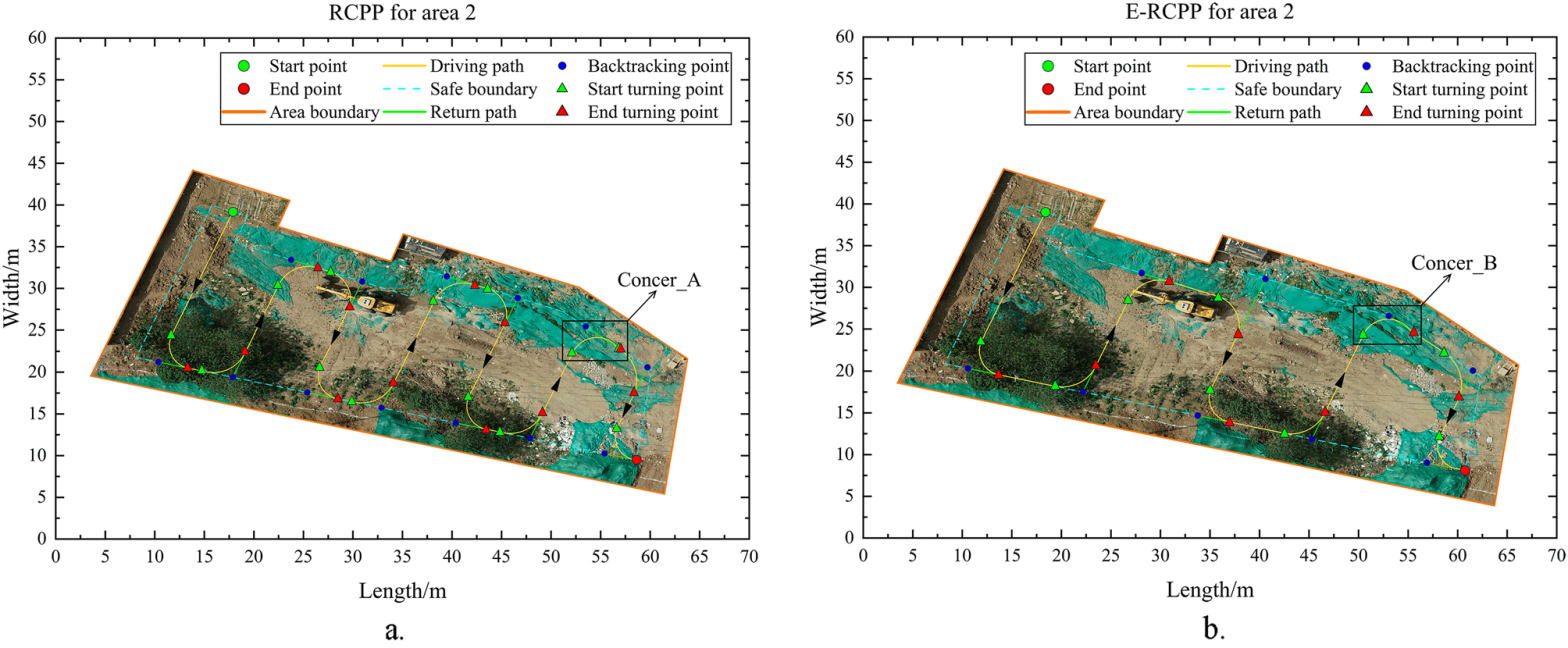
(**a**) Path generated by RCPP in area 2. (**b**) Path generated by E-RCPP in area.

**Figure 18:**
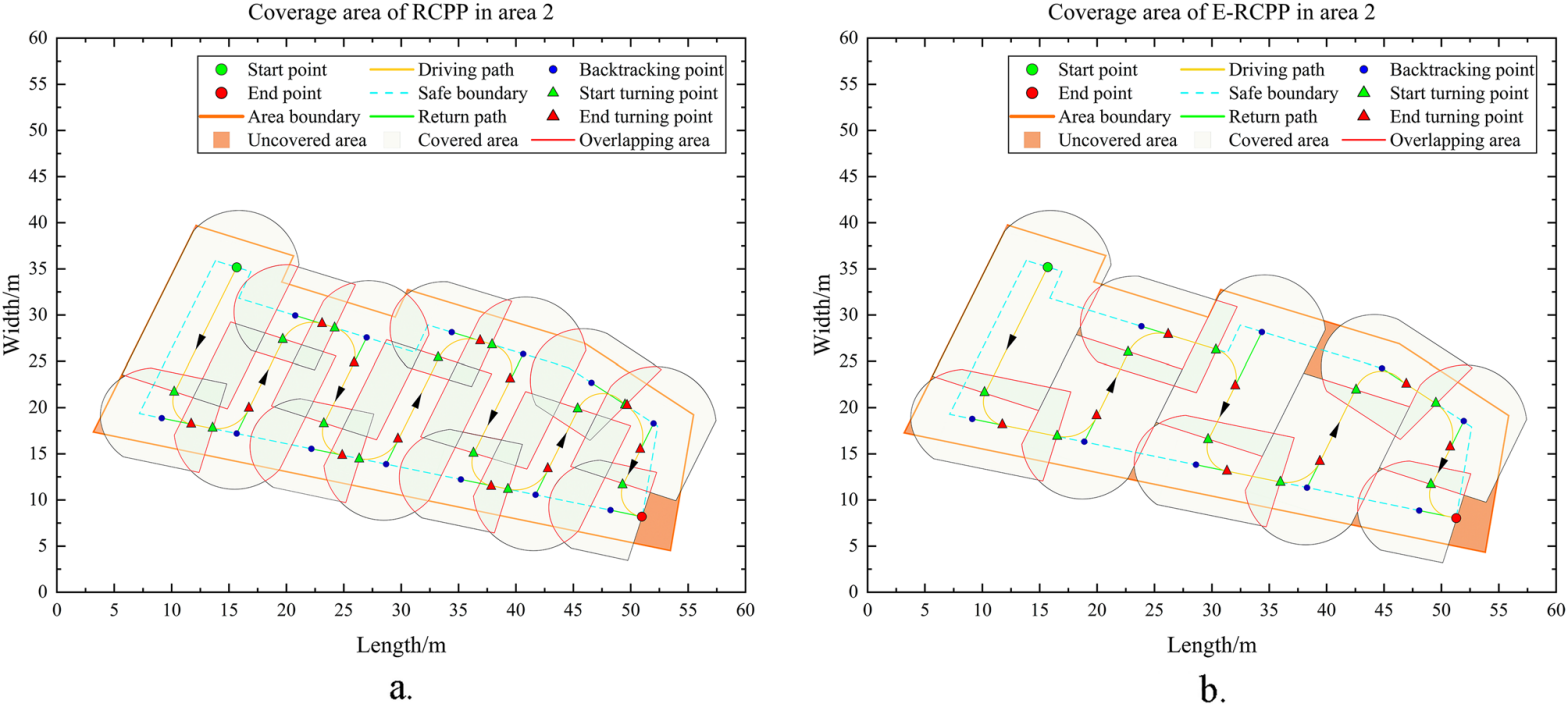
(**a**) Coverage of area 2 by the path generated by RCPP. (**b**) Coverage of area 2 by the path generated by E-RCPP.

It is worth noting that the coverage rate of the paths planned by the RCPP algorithm and E-RCPP algorithm hasn’t reach 100%. From Fig. 16a and b, it can be seen that there are some uncovered areas on the upper left of area 1 and scattered uncovered areas at the lower left, which is mainly due to the setting of the safe boundary. When the excavator approaches the safe boundary during driving, it will trigger the turning operation or drive along the safe boundary without exceeding the boundary range, which causes that some corners cannot be completely covered. In fact, when using BFP to deal with complete coverage path planning for convex areas, the coverage problem at the corners is difficult to avoid because the construction area is often not a positive polygon, but has many corners with different angles. The excavator needs a safe circular construction area to ensure its construction and turning, but when it is too close to the corner, the radius of the common tangent circle decreases sharply. However, the actual coverage rate should be slightly higher than the coverage rate shown in Table 4, because the ending point of the excavator is always located on the safe boundary, and the excavator often drives in reverse during the construction, which also results in insufficient coverage at the end point. In fact, the excavator still conducts earthwork operation when it leaves Area 1 from the end point, so the actual area of uncovered area is slightly lower than the theoretical calculation value.

#### Area 2

Different from Area 1, Area 2 is a non-convex area, it need to consider whether to decompose it into multiple convex sub-areas when planning a complete coverage path. In fact, Area 2 is a special non-convex area that satisfies the non-decomposition principle proposed in Sect. “Decomposition of construction area”, so it can plan directly. Figure 17a and b show the paths generated by the RCPP algorithm and E-RCPP algorithm in Area 2. It can be seen that after being adjusted by the adaptive spacing adjust model, the spacing of each parallel path expands significantly, from the initial setting of 8 m directly to the maximum spacing of 11.767 m, this phenomenon leads to a significant decrease in the number of turns as well as the overlap rate, which is very beneficial for the excavator's driving, and can easily cause a decrease in the coverage rate. Figure 18a and b show the coverage area of the path generated by the RCPP algorithm and the E-RCPP algorithm in Area 2, it can see that compared with the RCPP algorithm, the coverage of the E-RCPP algorithm decreases not only occur at the end point, but also at the middle of the path, which is caused by the increase in path spacing. The construction area of the excavator along the path is a combination of multiple semicircles and rectangles, this special combination requires the spacing between parallel paths to be kept within a relatively reasonable range, too low will lead to the reduction of construction efficiency, while too high will lead to the decline of coverage. This study also takes this into consideration when designing the cost function, specifically, the increase in coverage rate brought about by the increase in turn times will make each item of the cost function in a state of mutual checks and balances, so as to obtain the optimal path. The E-RCPP algorithm achieves a coverage rate of 97.51%, and considering that the area at the end point can still be covered, the actual coverage will be larger, this performance to be acceptable. In fact, the E-RCPP algorithm is very effective in adjusting the spacing of parallel path in narrow and long area such as Area 2. The phenomenon of too many turns and high overlap rate due to unreasonable initial spacing settings will be effectively improved after adjusting with the adaptive spacing adjust model, but this is actually a rather special case. In general, the planning strategy for narrow areas is to generate BFP parallel to one of the long edges, which will have fewer turns. The reason why such simple planning strategy are not applicable to Area 2 is that the existence of depressions on one of the long edges of Area 2 makes the parallel paths too far away from the outer edge of this side, which will cause a significant decrease in coverage rate.

It is worth noting that when the excavator turns at corner A in Fig. 17a and corner B in Fig. 17b, the turning situation here is different from other corners since the turning radius of the excavator is larger than the radius of the common tangent circle of the adjacent boundary at the corner. According to the turning method proposed in Sect. “Corner problem”, the driving trajectory of the excavator will slightly exceed the safe boundary of the construction area when making a turn here, this phenomenon does not affect the normal construction operation of the excavator, because the safe boundary is determined according to its construction characteristics, rather than driving characteristics, the former value is often much larger than the latter.

#### Area 3

**Figure 19:**
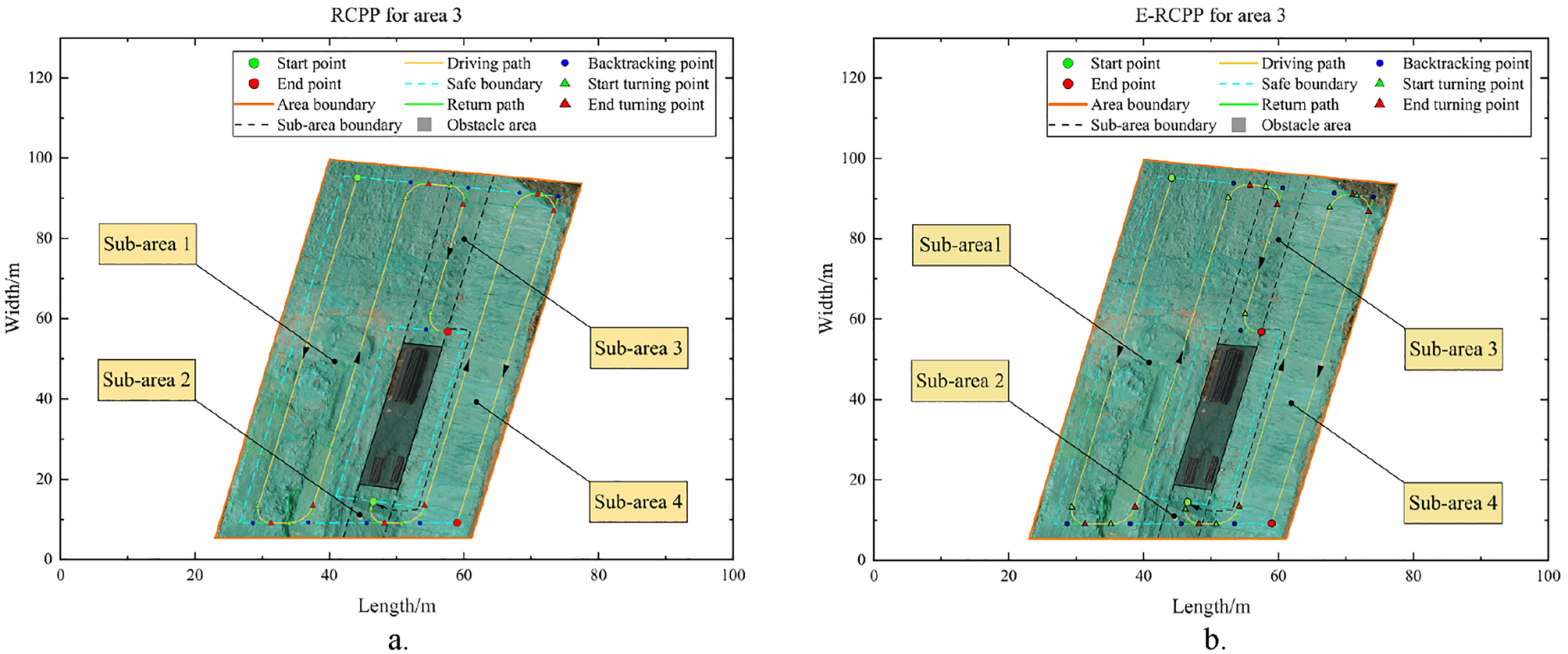
(**a**) Path generated by RCPP in area 3. (**b**) Path generated by E-RCPP in area 3.

**Figure 20:**
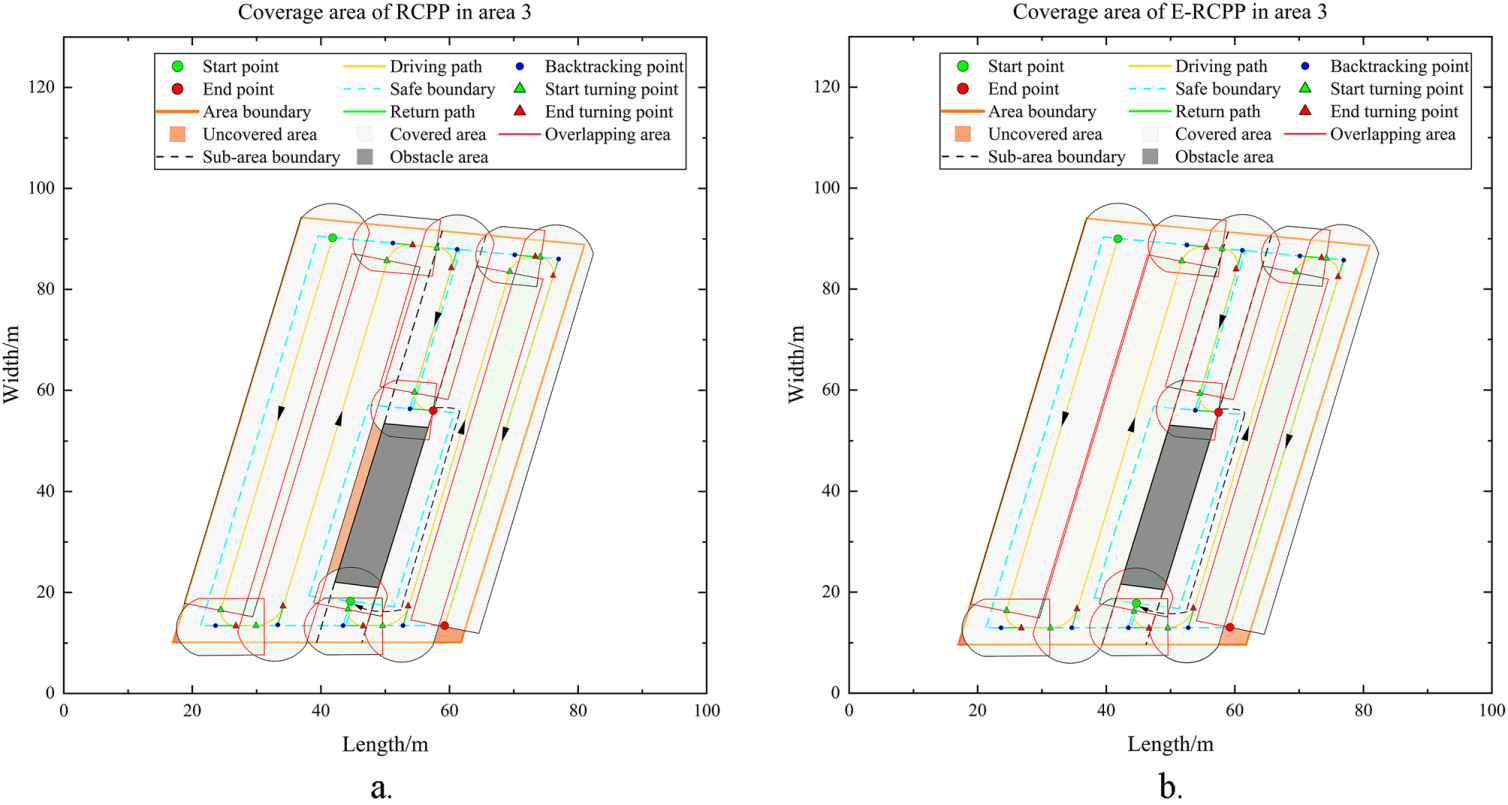
(**a**) Coverage of area 3 by the path generated by RCPP. (**b**) Coverage of area 3 by the path generated by E-RCPP.

Area 3 is also a convex area, it differs from Area 1 and Area 2 in that there is an obstacle with an area of 252.6 m^2^ inside Area 3. Taking the direction perpendicular to the long side of the area as the scanning direction, the Area 3 is decomposed using the BCD method to obtain four convex sub-areas, which are marked in Fig. 19a and b, respectively. Using the decomposed sub-areas to plan separately, and then connecting these sub-paths, the path planning of Area 3 can be realized. By comparing Fig. 19a and b, it can find that the path length and the number of turns generated by the RCPP algorithm and the E-RCPP algorithm are very close, mainly because the area of the sub-areas obtained after decomposition are small and only a few turns are needed to cover the sub-areas, which makes the adaptive spacing adjustment model only fine-tune the spacing and has little impact on the path length and the number of turns. The E-RCPP algorithm still superior to the RCPP algorithm in terms of coverage rate and overlap rate. Figure 20a and b show the coverage areas of the paths generated by the RCPP algorithm and the E-RCPP algorithm. It can be seen that the increase of coverage rate and the decrease of overlap rate are mainly reflected in sub-area 1, which is actually easy to understand because the relatively large area of sub-area 1 gives the adaptive spacing adjustment model a certain adjustment space, which makes the path spacing in sub-area 1 adjusted to a more reasonable value and thus achieves optimization. It is worth noting that the connecting line between sub-area 2 and sub-area 3 only represents the trajectory of the excavator and no earthwork is carried out in this section of road, so this additional section of the path will not affect the cost function of the E-RCPP algorithm.

## Conclusion

The earthwork construction has the characteristics of large scale, strong repeatability and high risk. In the traditional earthwork construction process, the construction personnel only rely on experience to operate the excavator, without the optimal path as a guide, which leads to strong randomness and low construction efficiency. The development of automated earthwork construction system for excavators is an ideal way to solve the above problems. The complete coverage path planning algorithm proposed in this study can be applied as one of the core technologies to develop this system, which can be used to assist remote or automated earthwork construction and ensure safety.

In the application of practical scenarios, the DOM of the construction area is established by UAV and imported into the control system of the autonomous excavator to generate corresponding commands and guide the construction process, which will help overcome the shortcomings of traditional excavators relying on manual operation, optimize the earthmoving construction process, and improve the efficiency and safety. The E-RCPP algorithm proposed in this study considers many related factors of construction, calculates the optimal path, and provides route guidance for earthwork. Case studies conducted in three different types of construction areas show that the E-RCPP algorithm proposed in this study has superior performance in terms of path length, number of turns, coverage rate and overlap rate of the planned paths compared to the conventional RCPP algorithm. The adaptive spacing adjustment model and turning strategy proposed in this study can adjust the spacing between parallel paths, reduce the influence of low coverage rate at corners caused by difficult turning operation. At the same time, this study also proposes a non-decomposition principle to deal with the complete coverage path planning problem in non-convex areas. The three experimental areas used in this study include convex area without obstacles, convex area containing obstacles, and non-convex areas without obstacles, for different shapes of construction areas, almost all of them can be decomposed into the above three areas by the BCD method, so this actually represents most types of construction areas. The experimental results demonstrate that the E-RCPP algorithm has better performance compared to the RCPP algorithm.

At present, the E-RCPP algorithm proposed in this study still has some limitations, mainly including the following: The earthwork needs to be transported by trucks, so how to determine the loading position of trucks and how to reserve a safe path for trucks still need to be further studied. Secondly, there are some moving objects in the earthwork construction environment, so it is very necessary to achieve dynamic obstacle avoidance. In addition, there is an inevitable skid phenomenon when the excavator is turning, which leads to the error between the theoretical route and the actual route. In future research work, the above problems will be continued explored, and some additional factors, including soil conditions, weather changes, etc. will be considered to further improve the algorithm.

## Data Availability

The datasets generated and analyzed during the current study are not publicly available due to the technical confidentiality of the funders, but are available from the corresponding author on reasonable request.

## References

[1] Luo Q (2021). Occupational health risk assessment based on dust exposure during earthwork construction. J. Build. Eng..

[2] Sluiter JK, de Croon EM, Meijman TF, Frings-Dresen MHW (2003). Need for recovery from work related fatigue and its role in the development and prediction of subjective health complaints. Occup. Environ. Med..

[3] Huang X, Hinze J (2006). Owner’s role in construction safety. J. Constr. Eng. Manag..

[4] Alshboul, O., Shehadeh, A., Tatari, O., Almasabha, G. & Saleh, E. Multiobjective and Multivariable Optimization for Earthmoving Equipment. J. Facil. Manag. (2022).

[5] Kim S, Seo J, Russell JS (2012). Intelligent navigation strategies for an automated earthwork system. Automat. Constr..

[6] Elshaboury N, Marzouk M (2020). Optimizing construction and demolition waste transportation for sustainable construction projects. Eng. Constr. Archit. Manag..

[7] Yeom D, Yoo H, Kim Y (2019). 3D surround local sensing system H/W for intelligent excavation robot (IES). J. Asian Archit. Build..

[8] Seo J, Lee S, Kim J, Kim S (2011). Task planner design for an automated excavation system. Automat. Constr..

[9] Kim J, Lee D, Seo J (2020). Task planning strategy and path similarity analysis for an autonomous excavator. Automat. Constr..

[10] Vu NT, Tran NP (2020). Path planning for excavator arm: Fuzzy logic control approach. J. Robot..

[11] Guo Y, Cui H, Li S (2022). Excavator joint node-based pose estimation using lightweight fully convolutional network. Automat. Constr..

[12] Jelavic, E. & Hutter, M., *in International Conference on Intelligent Robots and Systems (IROS)* (Macau, China, 2019), pp2292.

[13] Seongcheol, W., Juneyeong, Y., Mingi, J., Il-Chul, M. & Jinkyoo, P., in *2018 IEEE International Conference on Systems, Man, and Cybernetics (SM*C) (Miyazaki, Japan, 2018), pp. 4236.

[14] Kim J, Lee S, Seo J, Lee D, Choi HS (2021). The integration of earthwork design review and planning using UAV-based point cloud and BIM. Appl. Sci..

[15] Xie S, Chu X, Zheng M, Liu C (2019). Ship predictive collision avoidance method based on an improved beetle antennae search algorithm. Ocean Eng..

[16] Luo, B., Huang, Y., Deng, F., Li, W. & Yan, Y., in *Asia-Pacific Conference on Image Processing, Electronics and Computers* (Dalian, 2021), p 316.

[17] Acar EU, Choset H, Rizzi AA, Atkar PN, Hull D (2002). Morse decompositions for coverage tasks. Int. J. Robot. Res..

[18] Kim *et al.*, in *International conference on control, automation and systems* (Seoul, 2014), p 730.

[19] Choset H (2000). Coverage of known spaces: The boustrophedon cellular decomposition. Auton. Robot..

[20] Zhou L, Wang Y, Zhang X (2020). Complete coverage path planning of mobile robot on abandoned mine land. Chin. J. Eng..

[21] Kim S, Russell JS, Koo K (2003). Construction robot path-planning for earthwork operations. J. Comput. Civil Eng..

[22] Guastella DC (2019). Complete coverage path planning for aerial vehicle flocks deployed in outdoor environments. Comput. Electr. Eng..

[23] Vasquez-Gomez JI, Marciano-Melchor M, Valentin L, Herrera-Lozada JC (2020). Coverage path planning for 2D convex regions. J. Intell. Robot. Syst..

[24] Sherafatm B (2020). Automated methods for activity recognition of construction workers and equipment: State-of-the-art review. J. Constr. Eng. Manage..

[25] Shehadeh A, Alshboul O, Tatari O, Alzubaidi MA, Salama AHES (2022). Selection of heavy machinery for earthwork activities: A multi-objective optimization approach using a genetic algorithm. Alex. Eng. J..

